# Co-ensiling corn silage with *Pleurotus*-based spent mushroom substrate and oilseeds: Impacts on rumen fermentation, digestibility, and methane emissions in a RUSITEC system

**DOI:** 10.3934/microbiol.2026008

**Published:** 2026-04-10

**Authors:** Chika C. Anotaenwere, Omoanghe S. Isikhuemhen, Peter A. Dele, Ahmed E. Kholif, Michael Wuaku, Oludotun O. Adelusi, Deborah O. Okedoyin, Joel O. Alabi, Kelechi A. Ike, John O. Adebayo, Kiran Subedi, Uchenna Y. Anele

**Affiliations:** 1 Department of Animal Sciences, North Carolina Agricultural and Technical State University, Greensboro, NC 27411, USA; 2 Department of Natural Resources and Environmental Design, North Carolina Agricultural and Technical State University, Greensboro, NC 27411, USA; 3 Department of Pasture and Range Management, Federal University of Agriculture, Abeokuta, Ogun State, Nigeria; 4 Dairy Science Department, National Research Centre, 33 Bohouth St. Dokki, Giza 12622, Egypt; 5 Analytical Services Laboratory at CAES, North Carolina Agricultural and Technical State University, Greensboro, USA

**Keywords:** spent mushroom substrate, oilseeds, methane mitigation, corn silage, greenhouse gases, fiber digestibility

## Abstract

The present study examined the effects of partially replacing corn silage (CS) with *Pleurotus*-based spent mushroom substrate (SMS) and oilseeds (OS) on rumen fermentation characteristics, nutrient digestibility, volatile fatty acid (VFA) profiles, and greenhouse gas emissions using a rumen simulation technique (RUSITEC). Four silages were evaluated: A control diet containing 100% CS and three co-ensiled treatments in which CS was partially replaced with *Pleurotus*-based SMS and OS. The oilseed fraction consisted of equal proportions of soybean and canola seeds. The treatments were: CS90 (90% CS + 5% SMS + 2.5% soybean seed + 2.5% canola seed), CS80 (80% CS + 10% SMS + 5% soybean seed + 5% canola seed), and CS60 (60% CS + 20% SMS + 10% soybean seed + 10% canola seed). Dry matter (DM), organic matter, and crude protein contents were not affected by treatment, whereas ash content increased (P < 0.001) with increasing SMS-OS inclusion. Increasing SMS-OS inclusion significantly reduced non-fiber carbohydrates, hemicellulose and cellulose, while ether extract, neutral detergent fiber, acid detergent fiber, and acid detergent lignin concentrations were increased (P < 0.05). Ruminal fermentation pH, effluent volume, and total gas production showed only numerical differences or trends. DM digestibility increased markedly with SMS-OS inclusion (P < 0.001), rising from 38.7% in the control to 53.0, 49.9, and 52.5% in CS90, CS80, and CS60, respectively, representing improvements of approximately 29–37%. In CS90, organic matter digestibility remained high (P < 0.001) and fiber digestibility was largely the same. Moreover, CS90 had the highest total VFA and acetate, whereas CS60 had the highest propionate proportion (P < 0.05). All SMS-OS diets substantially reduced methane production (P = 0.003) and carbon dioxide emissions (P = 0.014) relative to the control. Methane output declined by approximately 60, 45, and 35% in CS90, CS80, and CS60, respectively (34.2–55.6 vs. 85.7 mg/g DM), while carbon dioxide emissions were reduced by about 49, 26, and 17% (134.7–218.4 vs. 264.0 mg/g DM). In conclusion, co-ensiling corn silage with SMS and oilseeds improved DM utilization while influencing methane production, with reductions observed in all the treatments compared with the control without adversely affecting rumen fermentation stability or fiber digestion under RUSITEC conditions.

## 1. Introduction

Meeting the rising global demand for animal-source foods while limiting the environmental footprint of livestock production remains a central challenge for ruminant systems. The global population is projected to reach approximately 9.7 billion by 2050, placing increasing pressure on feed resources, arable land, and the climate system [1]. Enteric methane (CH_4_) produced during ruminal fermentation is a major source of agricultural greenhouse gas emissions and represents a substantial fraction of global anthropogenic CH_4_ emissions [2]. Consequently, reducing enteric CH_4_ emissions is a cornerstone of climate-smart livestock strategies. Among the available mitigation approaches, nutritional interventions are considered particularly attractive because they are practical, cost-effective, and readily applicable at the farm level [3]. In parallel, circular bioeconomy frameworks emphasize the recycling of agro-industrial by-products into animal feeds as a means of reducing waste streams and dependence on primary crops [4].

Spent mushroom substrate (SMS), the residual biomass remaining after mushroom cultivation, is generated in large quantities worldwide. When not utilized, SMS presents environmental and logistical challenges due to its high organic content and bulkiness [5]. Nevertheless, SMS typically contains residual proteins, carbohydrates, minerals, fungal biomass, and a partially modified lignocellulosic matrix, making it a potentially valuable feed or silage component when appropriately processed. In addition to residual plant material, SMS contains fungal cell wall components such as chitin and β-glucans as well as lignocellulolytic enzymes produced during fungal growth, which may partially degrade lignin, thereby increasing the accessibility of structural carbohydrates [6]. These characteristics can facilitate microbial colonization and ruminal fermentation, potentially enhancing fiber utilization and altering ruminal fermentation pathways [7],[8] Studies have shown that SMS derived from white-rot fungi, such as *Pleurotus* spp., retains considerable nutritive value and can partially replace conventional forages in ruminant diets [9],[10]. Biological pretreatments and ensiling have been particularly effective in improving fiber degradability and nutrient availability from SMS [11]. *In vitro* work by Anotaenwere et al. [11] demonstrated that co-ensiling *Pleurotus*-based SMS with corn silage (CS) reduced CH_4_ and CO_2_ production, with the extent of mitigation dependent on ensiling duration. Complementarily, an *in vivo* study by Rangubhet et al. [12] reported that partial replacement of forage with SMS-based silages decreased CH_4_ emissions and altered ruminal protozoal populations in cattle.

Dietary lipids supplied by oil-rich seeds represent another well-established strategy for mitigating enteric CH_4_ emissions. Oilseeds such as canola and soybean provide unsaturated fatty acids that can inhibit methanogenic archaea and protozoa and serve as alternative hydrogen sinks through biohydrogenation, shift ruminal fermentation toward propionate, and, at higher inclusion levels, potentially depress fiber digestion [13]. In addition, lipids may suppress ruminal methanogenesis by reducing protozoal populations, altering microbial community structure, and decreasing fermentable carbohydrate availability for hydrogen production [14]. Studies have demonstrated that supplementing ruminant diets with plant oils or high-oil coproducts can substantially reduce CH_4_ emissions without compromising animal performance when used at moderate inclusion rates [7],[15]. These observations suggest that combining SMS with oilseeds may yield complementary effects: Fungal modification of the lignocellulosic matrix could enhance fiber accessibility and silage quality, while lipids would exert a direct antimethanogenic effect [7]. Co-ensiling these components with CS may therefore result in nutritionally adequate feeds with improved environmental performance. Despite increasing interest in the use of SMS and oilseeds as alternative feed resources, information remains limited on how different inclusion levels of these materials, when co-ensiled with CS, affect rumen fermentation, nutrient digestibility, volatile fatty acids (VFAs) profiles, and greenhouse gas emissions.

Therefore, our objective was to evaluate the long-term effects of partially replacing CS with *Pleurotus*-based SMS and oilseeds on rumen fermentation characteristics, nutrient digestibility, VFA production, and greenhouse gas emissions using a rumen simulation technique (RUSITEC). This experiment was designed as a long-term follow-up to our previous short-term *in vitro* study [7] to validate the persistence of the observed responses under sustained *in vitro* fermentation conditions. We hypothesized that co-ensiling CS with increasing proportions of SMS and oilseeds would (i) maintain a stable rumen fermentation environment, (ii) enhance or at least preserve dry matter (DM) and fiber digestibility, and (iii) substantially reduce CH_4_ and CO_2_ emissions compared with sole CS. This approach could therefore represent a viable strategy for valorizing agro-industrial residues within climate-smart ruminant production systems.

## 2. Materials and methods

### 2.1. Ethical approval of the study

The North Carolina Agricultural and Technical State University's Institutional Animal Care and Use Committee (IACUC) approved all animal procedures used in this study (Protocol ID: LA21-009). The study was conducted at the Beef Research and Training Facility (BRTF) in Greensboro, NC. Daily health checks were performed on the cannulated cows by the BRTF, and treatment was administered in accordance with standard management practices following protocol LA21-009. All procedures complied with institutional and national guidelines for the care and use of agricultural animals in research.

### 2.2. Silage preparation

Silage was prepared according to the method detailed by Anotaenwere et al. [7]. Corn was harvested manually from the university farm, leaving a 1 cm stubble, when the kernels were approximately one-third milk-line maturity. The harvested corn was then chopped using a forage chopper into approximately 2 cm pieces to facilitate proper packing and optimal silage fermentation. The SMS used in this study was from corn stover (100% of the growth medium) and was produced at the university mycology laboratory. Soybean and canola seeds, used as oilseeds (OS), were purchased from Nutrient Ag Solutions, Brown Summit, Greensboro, NC, USA. The mixture of corn, SMS, and OS was ensiled in four 5-L plastic cylindrical buckets (35 cm high and 16 cm in diameter) per treatment, with each bucket serving as a mini silo. Silage was packed to an approximate density of 600 g DM per liter (equivalent to ~ 600 kg fresh matter per m^3^) using a hand tamper to ensure firm compaction. This packing density was critical for effective oxygen exclusion, thereby promoting desirable anaerobic fermentation, regulating microbial activity, and improving silage quality. Ensiling was done within 6 h after harvest to limit plant respiration and undesirable microbial activity with minimal disturbance during storage, and the buckets were stored at room temperature (20 ± 2 °C) for 42 days. Silo weights were recorded before filling and after opening to monitor DM recovery. Upon opening, silages were thoroughly mixed within each treatment, and representative subsamples were collected for chemical analyses. It should be noted that measurements of DM recovery, silage fermentation profile (pH and organic acids), and ammonia-N, as silage quality assessments, were not conducted in this study. Consequently, the assessment of silage quality was based on chemical composition, nutrient digestibility, VFAs, and greenhouse gas emissions.

### 2.3. Study design and chemical analysis

A completely randomized design with four replicates per treatment was used in this study. Each replicate consisted of an independent RUSITEC fermenter, and measurements were taken daily over a 5-day sampling period following a 4-day adaptation phase. This repeated-measures approach increased data reliability and partially compensated for the limited number of independent fermenters. The treatments included sole CS (used as the control), 90% CS + 5% SMS + 5% OS (CS90), 80% CS + 10% SMS + 10% OS (CS80), and 60% CS + 20% SMS + 20% OS (CS60). All mixtures were ensiled for 42 days prior to use. These dietary treatments were selected based on other findings [7]. The low (5% SMS + 5% OS), medium (10% SMS + 10% OS), and high (20% SMS + 20% OS) inclusion levels were chosen based on a combination of previous *in vitro* findings indicating effective CH_4_ and CO_2_ mitigation, nutritional constraints ensuring comparable protein and energy content, preliminary trials assessing fiber digestibility and silage handling, and practical feeding scenarios reflecting realistic on-farm application. Therefore, in this study, treatments with low, medium, and high inclusion levels of SMS and OS were chosen for further testing using the Rumen Simulation Technique (RUSITEC). These levels were considered based on a balance of improved ruminal fermentation, fiber utilization, and decreased greenhouse gas emissions observed in the earlier batch culture experiment. The diets were analyzed using the standard methods of AOAC [16] for DM (#930.15), ether extract (EE; #920.39), ash (#942.05), and nitrogen (N; #954.01). Unless otherwise stated, all chemical composition data are expressed on a DM basis. Organic matter (OM) was calculated by subtracting ash content from DM and expressed as a percentage. Neutral detergent fiber (NDF) was determined according to the procedure of Van Soest et al. [17], while acid detergent fiber (ADF; #973.18) was analyzed using the ANKOM 200 Fiber Analyzer (ANKOM Technology Corporation, Fairport, NY, USA). Acid detergent lignin (ADL) was measured by solubilizing cellulose with concentrated sulfuric acid, following the analytical protocols provided by ANKOM Technologies.

### 2.4. RUSITEC fermentation

The *in vitro* rumen fermentation was conducted using a Rumen Simulation Technique (RUSITEC) system, which is a semi-continuous culture system designed to mimic rumen conditions under controlled laboratory settings. The RUSITEC system consisted of two identical fermenters, each with 8 chambers (n = 16), featuring 1000 mL fermentation vessels. These were randomly assigned to four groups, each with four replicates corresponding to the four dietary treatments. Each vessel was equipped with an inlet for infusing artificial saliva and an outlet for effluent. Although the number of independent fermenters per treatment was limited (n = 4), daily sampling over a 5-day collection period following a 4-day adaptation phase provided repeated measures that enhanced data reliability. At the experiment's onset, 700 mL of rumen fluid and 200 mL of artificial saliva, prepared following McDougall's buffer recipe [NaHCO_3_: 9.83 g/L, Na_2_HPO_4_: 3.69 g/L, KCl: 0.60 g/L, NaCl: 0.47 g/L, (NH_4_)_2_SO_4_: 0.30 g/L, MgCl_2_.6H_2_O: 0.061 g/L, CaCl_2_.2H_2_O: 0.0293 g/L] were introduced into each fermentation vessel. The initial pH levels of the rumen fluid and artificial saliva were 6.61 and 8.54, respectively, which were within the physiological range for rumen conditions and McDougall's buffer.

Three rumen-cannulated Black Angus beef cows (weight: 550 ± 10 kg) served as rumen inoculum donors for the RUSITEC fermenters. The cows had ad libitum access to water and pasture, primarily consisting of forage (over 99% hay and grass) and a mineral supplement of less than 1%. Ruminal contents were collected from the dorsal, ventral, and cranial regions of the cannulated cows to obtain a representative sample of the rumen environment. The rumen fluid from all three cows was combined, strained through four layers of cheesecloth, and transported in a pre-warmed, insulated Thermoflask to the laboratory to preserve microbial activity and temperature. On the first incubation day, approximately 50 g of rumen solids were added to each vessel and removed after 24 h, which facilitated microbial attachment and adaptation to the *in vitro* system. Sample bags containing the substrate were placed in the vessels for a 48-h ruminal fermentation period, reflecting a typical ruminal retention time for solid feed particles. The fermenters were submerged in a 39 °C water bath, maintaining a constant temperature similar to that of the bovine rumen, with continuous infusion of artificial saliva maintained at a rate of 21 rpm using a Watson-Marlow Pump 205U (Watson-Marlow Ltd., Cornwall, England). The experiment lasted 9 days, comprising 4 days for adaptation and 5 days for data collection. The 4-day adaptation period was applied to allow the microbial population to adjust to the corn silage-based experimental diets, stabilizing ruminal fermentation before data collection. This approach mitigated major discrepancies, though some differences from silage-adapted microbiota may have remained.

### 2.5. Fermentation measurements

During the 5-day sampling period, the total gas produced was collected daily in a Tedlar® gas sampling bag (Supelco®, Bellefonte, PA, USA) connected to the effluent flasks. Daily gas pressure readings were taken (Gas Flowmeter DM3, Alexander Wright Ltd., London, UK), with gas production expressed in mL/d. The composition of the gases produced, including CH_4_, CO_2_, ammonia (NH_3_), and hydrogen sulfide (H_2_S), was estimated from the effluent flasks using a portable gas analyzer (Biogas 5000, Landtec, Dexter, MI, USA) equipped with internal electrochemical and dual-wavelength infrared cells with a reference channel. Following calibration according to the manufacturer's instructions, gas readings were obtained by connecting the analyzer to the gas collection opening of each effluent flask. To ensure accuracy, the unit was purged between each sampling to remove any residual gas from the previous measurement.

The volume of effluent was measured daily at the time of feed bag exchange using a graduated cylinder, and pH values were determined immediately (Fisherbrand™ FE150 pH benchtop meter, Fisher Scientific, Waltham, MA, USA). Thereafter, 25 mL of liquid effluent was collected into a 50 mL Eppendorf tube containing 5 mL of diluted H_2_SO_4_ (72%) for NH_3_-N analysis [16]. Another 15 mL of liquid effluent was collected, preserved with 3 mL of 25% (w/w) metaphosphoric solution, and immediately frozen at −20 °C until it was needed for VFA determination. The VFA concentrations were quantified using gas chromatography (Agilent 7890B GC system with a Flame Ionization Detector, 7693 autosampler, Agilent Technologies, Santa Clara, CA, USA) and a capillary column (Zebron ZB-FFP, Phenomenex Inc., Torrance, CA, USA) following standard procedures published by Olagunju et al. [18]. This procedure enabled the quantification of total VFA concentration and the individual VFA profile. Overall, 20 individual replicate samples were collected for each treatment (4 vessels × 5 sampling days).

### 2.6. Nutrient digestibility

Following the same sampling period as described above, sample bags were removed from each fermenter after 48 h of *in vitro* fermentation, washed with cold water until the water ran clear, and oven-dried at 55 °C for 72 h. The residue weight was used to estimate DM digestibility (DMD). DMD (%) was calculated as: DMD = [(DM incubated − DM residue)/DM incubated] × 100. A known amount of residue from each sample was burned at 550 °C for 3 h in crucibles and weighed to determine OM digestibility (OMD). OMD (%) was calculated using OM values. The residues in each bag were analyzed for fiber components (NDF, ADF, and ADL) using the Ankom Fiber Analyzer (ANKOM Technology, Macedon, NY, USA) to calculate their degradability (NDFD, ADFD, and ADLD, respectively). Fiber degradability was expressed as the proportion of the respective fiber fraction that disappeared during the 48-h incubation.

### 2.7. Statistical analysis

The Shapiro–Wilk test was used to assess the normal distribution of variables based on model residuals. All variables exhibited a normal distribution. The data were analyzed using a completely randomized design in SAS (version 9.4; SAS Institute Inc., Cary, NC, USA) with the GLM procedure. Each replicate consisted of an independent RUSITEC fermenter and was considered the experimental unit. Measurements collected daily over the 5-day sampling period were averaged for each vessel prior to statistical analysis to avoid pseudoreplication. Therefore, the statistical analysis was based on four independent observations per treatment rather than treating daily measurements as independent replicates. Treatment was included as a fixed effect in the model. Means of significant variables were separated at P ≤ 0.05 using the Tukey multiple range test. The statistical model employed was Y_ij_=µ + T_i_ + _eij_, where Y_ij_ is the dependent variable, µ is the overall mean, T_i_ is the treatment effect, and e_ij_ is the residual error.

## 3. Results

### 3.1. Chemical composition of experimental silages

**Figure 1.**
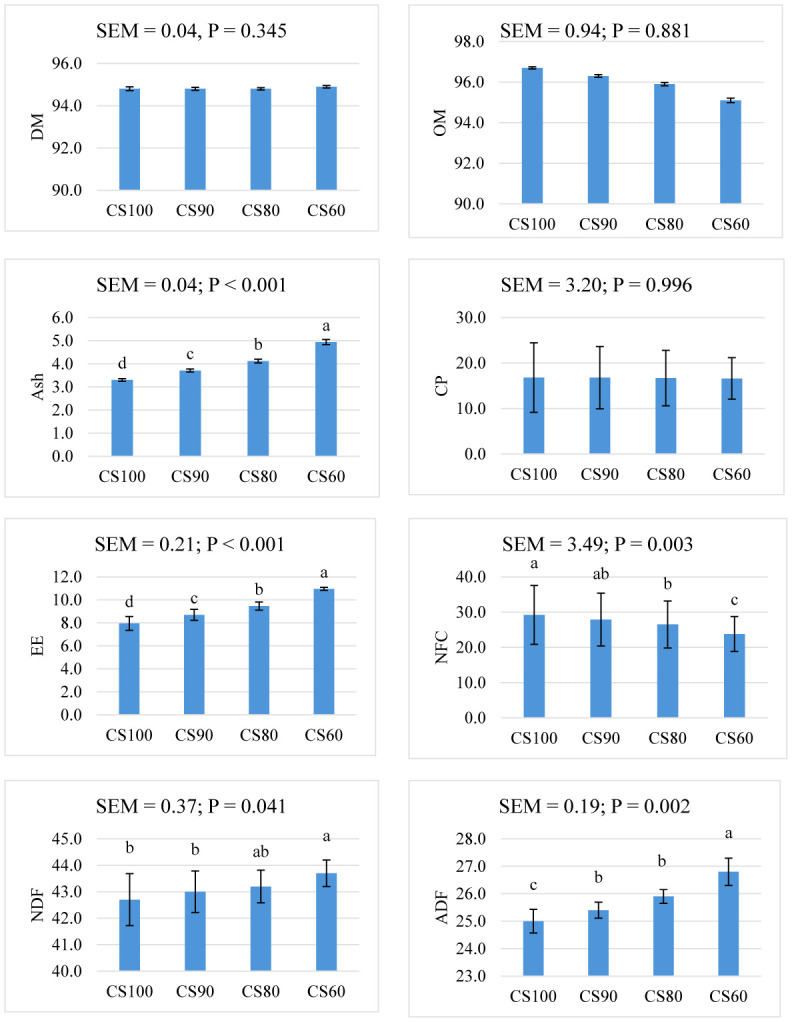
Chemical composition (% of DM) of control corn silage and corn silage co-ensiled with spent mushroom substrate (SMS) and oilseeds (OS). DM = dry matter; OM = organic matter; CP = crude protein; EE = ether extract; NFC = non-fiber carbohydrates; NDF = neutral detergent fiber; ADF = acid detergent fiber; ADL = acid detergent lignin. ^1^Treatments: Control = 100% CS; CS90 = 90% CS + 5% SMS + 5% OS; CS80 = 80% CS + 10% SMS + 10% OS; CS60 = 60% CS + 20% SMS + 20% OS.

The chemical composition of the control corn silage and the SMS–OS co-ensiled silages is shown in Figure 1. DM, OM, and crude protein (CP) contents were not affected by treatment and remained stable at 94.8–94.9% DM, 95.1–96.7%, and 16.6–16.8% DM, respectively. Ash content increased markedly (P < 0.001), rising from 3.30% DM in the control to 3.71 (+12%), 4.12 (+25%), and 4.94% DM (+50%) in CS90, CS80, and CS60, respectively. Ether extract content increased sharply with SMS–OS inclusion (P < 0.001), from 7.96% DM in the control to 8.70 (+9%), 9.46 (+19%), and 10.96% DM (+38%) in CS90, CS80, and CS60, respectively. In contrast, non-fiber carbohydrates decreased significantly (P = 0.003), declining from 29.2% DM in the control to 27.9 (−4%), 26.5 (−9%), and 23.8% DM (−19%).

NDF increased significantly (P = 0.041), from 42.7% DM in the control to 43.0 (+0.7%), 43.2 (+1.2%), and 43.7% DM (+2.3%) in CS90, CS80, and CS60, respectively. Acid detergent fiber also increased (P = 0.002), from 25.0% DM in the control to 25.4 (+2%), 25.9 (+4%), and 26.8% DM (+7%). Acid detergent lignin showed the largest proportional change (P < 0.001), increasing from 2.38% DM in the control to 3.06 (+29%), 3.74 (+57%), and 5.11% DM (+115%) in CS90, CS80, and CS60, respectively. Hemicellulose (P < 0.001) and cellulose (P = 0.005) contents declined significantly, with reductions of up to 4% relative to the control.

### 3.2. pH, volume of effluent, gas production, and digestibility

Ruminal fermentation pH numerically differed among treatments and remained within a narrow range (7.51–7.64) compared with the control (Table 1). In addition, effluent volume ranged from 527.5 to 565.0 mL/d, with no significant differences among treatments (P = 0.137), and effluent output increased relative to the control by about 3% in CS90, 2% in CS80, and 7% in CS60. Moreover, total gas production ranged from 2541 to 2876 mL/d and was not significantly affected by diet (P = 0.374). Regarding nutrient digestibility, OMD decreased progressively with SMS–OS inclusion (P < 0.001), declining from 97.4% in the control to 97.0% (−0.4%), 96.6% (−0.8%), and 95.3% (−2.2%) in CS90, CS80, and CS60. In contrast, DMD increased markedly (P < 0.001), rising from 38.7% in the control to 53.0% (+37%) in CS90, 49.9% (+29%) in CS80, and 52.5% (+36%) in CS60.

**Table 1.** Effects of partial replacement of corn silage (CS) with spent mushroom substrate (SMS) and oilseeds (OS) on *in vitro* fermentation pH, effluent volume, and *in vitro* nutrient digestibility.

Treatment^1^	pH	Volume of effluent (mL/d)	Gas volume (mL/d)	OMD (%)	DMD (%)
Control	7.64	527.5	2876	97.4^a^	38.7^b^
CS90	7.55	542.9	2541	97.0^a^	53.0^a^
CS80	7.51	538.4	2838	96.6^b^	49.9^a^
CS60	7.59	565.0	2608	95.3^c^	52.5^a^
SEM	0.093	15.75	166.6	0.15	1.62
P-value	0.111	0.137	0.374	<0.001	<0.001

^a,b^ Within a column, means with different superscripts differ (P < 0.05). OMD: Organic matter digestibility, DMD: Dry matter digestibility; SEM = standard error of the mean. ^1^Treatments: Control = 100% CS; CS90 = 90% CS + 5% SMS + 5% OS; CS80 = 80% CS + 10% SMS + 10% OS; CS60 = 60% CS + 20% SMS + 20% OS.

### 3.3. Volatile fatty acid profile

Total VFA concentrations were affected by diet (P = 0.036), ranging from 42.4 mmol/L in the control to 45.6 (+8%), 41.2 (−3%), and 36.2 mmol/L (−15%) in CS90, CS80, and CS60, respectively (Table 2). Moreover, the molar proportion of acetate declined with increasing SMS–OS inclusion (P = 0.037), decreasing from 62.1% in the control to 55.6% (−10%) in CS60. In contrast, propionate proportion increased (P = 0.012) from 18.7% in the control to 23.5% (+26%) in CS60. As a result, the acetate: Propionate ratio was significantly affected by diet (P = 0.041), increasing by approximately 12% in CS90 (3.83 vs. 3.42 in the control) and decreasing by 29% in CS60 (2.43). Moreover, butyrate proportion was not affected by treatment (P = 0.527).

Regarding minor VFAs, valerate proportion differed significantly among treatments (P = 0.013), decreasing by 16% in CS90 relative to the control, but increasing by approximately 16% and 20% in CS80 and CS60. On the other hand, isobutyrate proportion was unaffected by diet (P = 0.610), whereas isovalerate proportion was significantly influenced by treatment (P = 0.0057), being 35% lower in CS90 and 41% higher in CS60 compared with the control.

**Table 2.** Effects of partial replacement of corn silage (CS) with varying proportions of spent mushroom substrate (SMS) and oilseeds (OS) on total concentration (mmol/L) and molar proportions (%) of volatile fatty acids (VFA).

Treatment^1^	Total VFA (mmol/L)	Acetate (A; %)	Propionate (P; %)	A:P ratio	Butyrate (%)	Valerate (%)	Isobutyrate (%)	Isovalerate (%)
Control	42.4^ab^	62.1^a^	18.7^ab^	3.42^a^	16.9	1.34^ab^	0.60	0.29^bc^
CS90	45.6^a^	63.6^a^	18.4^b^	3.83^a^	16.8	1.12^b^	0.69	0.19^c^
CS80	41.2^ab^	59.2^ab^	18.7^ab^	3.39^a^	19.6	1.55^a^	0.63	0.31^ab^
CS60	36.2^b^	55.6^b^	23.5^a^	2.43^b^	18.1	1.61^a^	0.77	0.41^a^
SEM	2.69	2.24	1.98	0.378	1.58	0.104	0.120	0.044
P-Value	0.036	0.037	0.012	0.041	0.527	0.013	0.611	0.006

^a,b,c^ Within a column, means with different superscripts differ (P < 0.05). A:P = acetate:propionate ratio; VFA = volatile fatty acids; SEM = standard error of the mean. ^1^Treatments: Control = 100% CS; CS90 = 90% CS + 5% SMS + 5% OS; CS80 = 80% CS + 10% SMS + 10% OS; CS60 = 60% CS + 20% SMS + 20% OS.

### 3.4. Greenhouse gas and ammonia emissions

CH_4_ production was strongly affected by diet (P = 0.003); compared with the control (85.7 mg/g DM), CH_4_ emissions were reduced by approximately 60% in CS90, 45% in CS80, and 35% in CS60 (Table 3). Similarly, carbon dioxide (CO_2_) production also differed among treatments (P = 0.014), with output reduced relative to the control (264.0 mg/g DM) by 49% in CS90, 26% in CS80, and 17% in CS60. In contrast, NH_3_ production was not significantly affected by diet (P = 0.351), although values were numerically lower than the control by approximately 36% in CS90, 15% in CS80, and 14% in CS60. Likewise, H_2_S production did not differ significantly among treatments (P = 0.254) but declined numerically by about 68%, 66%, and 80% in CS90, CS80, and CS60, respectively, compared with the control.

**Table 3.** Effects of partial replacement of corn silage (CS) with varying proportions of spent mushroom substrate (SMS) and oilseeds (OS) on productions of CH_4_, CO_2_, NH_3_, and H_2_S.

Treatment^1^	CH_4_ (mg/g DM incubated)	CO_2_ (mg/g DM incubated)	NH_3_ (mmol/g DM incubated)	H_2_S (mmol/g DM incubated)
Control	85.7^a^	264.0^a^	633.2	5765
CS90	34.2^c^	134.7^b^	405.6	1830
CS80	47.5^bc^	194.4^ab^	538.7	1971
CS60	55.6^b^	218.4^a^	546.1	1137
SEM	6.86	29.41	112.32	1789
P-value	0.003	0.014	0.3505	0.2535

^a,b^ Within a column, means with different superscripts differ (P < 0.05). CH_4_ = methane, CO_2_ = carbon dioxide, NH_3_ = ammonia, H_2_S = hydrogen sulfide; SEM = standard error of the mean. ^1^Treatments: Control = 100% CS; CS90 = 90% CS + 5% SMS + 5% OS; CS80 = 80% CS + 10% SMS + 10% OS; CS60 = 60% CS + 20% SMS + 20% OS.

### 3.5. Fiber digestibility

Table 4 shows the effects of treatments on fiber digestibility. NDFD was not affected by treatment (P = 0.632) and remained within ±1% of the control value (64.8%). In contrast, ADFD tended to decline with increasing SMS-OS inclusion (P = 0.086), decreasing by approximately 2–3% relative to the control. Moreover, ADLD was significantly influenced by diet (P = 0.037), increasing by about 6% in CS90 and decreasing by roughly 5% in CS80 compared with the control, while CS60 showed values similar to the control.

**Table 4.** Effects of partial replacement of corn silage (CS) with varying proportions of spent mushroom substrate (SMS) and oilseeds (OS) on fiber digestibility (%).

Treatment^1^	NDFD (%)	ADFD (%)	ADLD (%)
CS100	64.8	56.4^a^	21.6^ab^
CS90	65.3	55.4^ab^	22.8^a^
CS80	64.3	55.3^ab^	20.6^b^
CS60	65.2	54.6^b^	21.5^ab^
SEM	0.63	0.49	0.72
P-Value	0.632	0.086	0.0367

^a,b^ Within a column, means with different superscripts differ (P < 0.05). NDFD: = neutral detergent fiber digestibility; ADFD = acid detergent fiber digestibility; ADLD = acid detergent lignin digestibility. ^1^Treatments: Control = 100% CS; CS90 = 90% CS + 5% SMS + 5% OS; CS80 = 80% CS + 10% SMS + 10% OS; CS60 = 60% CS + 20% SMS + 20% OS.

## 4. Discussion

We acknowledge that we employed four replicates per treatment, which is lower than in some mini-silo studies that entailed six or more replicates to increase statistical robustness. This limited replication may have reduced the power to detect subtle but biologically relevant differences, particularly for ruminal fermentation parameters with high variability. Nevertheless, the repeated daily sampling over five days and consistent directional trends across treatments provided confidence in the observed major effects of diet on *in vitro* fermentation. While the repeated sampling over multiple days improved data reliability, the findings should be interpreted with caution, as *in vitro* results may not fully replicate *in vivo* rumen dynamics. Future studies are warranted to validate these responses under practical feeding conditions in live animals. It should be noted that the rumen inoculum was derived from hay- and grass-fed donors; while the adaptation period in the RUSITEC system mitigates the impact, the microbial community may differ from that of silage-adapted animals. This discrepancy could have influenced fiber degradation, ruminal fermentation patterns, and gas production, representing a limitation of the experimental design that should be considered when extrapolating the results to *in vivo* silage-based feeding scenarios.

### 4.1. Chemical composition of silages

Co-ensiling CS with SMS and oilseeds modified diet composition in ways that help explain the *in vitro* fermentation and digestibility responses observed in the RUSITEC. Although DM, OM, and CP concentrations were unaffected, SMS-OS inclusion significantly reduced NFC, hemicellulose, and cellulose, while increasing ash, EE, NDF, ADF, and ADL. The increase in ash content with SMS-OS inclusion likely reflects the higher mineral concentration of the SMS and OS compared with the control CS, which is consistent with their reported nutrient composition. The compositional changes were largely additive, reflecting the intrinsic chemical characteristics of SMS and oilseeds. Notably, EE and ADL increased progressively with higher SMS-OS inclusion in the diets. This shift toward higher lipid and lignin contents, alongside lower readily fermentable carbohydrates, provides an important framework for interpreting the largely stable ruminal fermentation conditions, enhanced DMD, and marked reductions in CH_4_ production [7]. The elevated ash content further reflects the mineral-rich nature of SMS, which has been reported to contain substantial concentrations of calcium, potassium, and phosphorus derived from fungal biomass and cultivation substrates.

### 4.2. pH, effluent output, gas production, and nutrient digestibility

Despite the compositional changes, *in vitro* fermentation pH remained within a narrow, physiologically suitable range (7.51–7.64) and was only modestly affected by treatment. The ruminal pH was slightly higher than *in vivo* values, reflecting the buffering capacity of the continuously infused artificial saliva. This pH range is physiologically suitable and maintains stable microbial activity, ensuring reliable measurement of fermentation and gas production under semi-continuous culture conditions. The lowest pH value, observed in CS80, was approximately 1.7% lower than that of the control and well within the optimal range for fibrolytic activity. Methanogenic archaea typically thrive between pH 6.2 and 7.8 [19], indicating that the substantial reductions in CH_4_ observed here were not driven by pH inhibition. This pH stability is characteristic of continuous culture systems such as RUSITEC, where constant buffering and removal of *in vitro* fermentation products prevent acid accumulation [20]. Similar neutral pH values have been reported by others using fungal-treated roughages [21],[22], supporting the compatibility of SMS-based diets with a stable ruminal environment.

Effluent volume showed numerical differences among treatments. In the RUSITEC system, effluent flow reflects dilution of ruminal fermentation products, microbial turnover, and water associated with the feed matrix [20]. The tendency toward greater effluent output with increasing SMS–OS inclusion may reflect altered physical properties of the feed matrix, including higher fiber content and water-holding capacity of fungal residues, rather than changes in ruminal fermentation intensity. Importantly, total gas production remained unaffected, indicating that overall fermentative activity was maintained across treatments. These findings suggest that SMS–OS inclusion altered liquid flow patterns without measurably affecting the extent of ruminal fermentation. Teoh et al. [23] showed that changes in CH_4_ production can occur without alterations in effluent volume, highlighting that gas mitigation can occur independently of total ruminal fermentation rate.

Digestibility responses were more pronounced. OMD declined progressively with increasing SMS–OS inclusion. This pattern is consistent with the higher lignin and lower NFC contents of the co-ensiled diets. Lignin is a major determinant of indigestible OM, forming physical and chemical barriers that limit microbial access to cell wall polysaccharides [24]. The absence of major reductions in OMD at low and moderate SMS–OS inclusion levels indicates that lignin-related constraints become nutritionally relevant only at the highest substitution rate. In this study, ADL more than doubled in CS60, and even modest increases in lignin can disproportionately reduce OM digestibility. Concurrently, NFC declined by up to 19%, reducing the pool of rapidly fermentable substrates that typically contributed to OM disappearance.

In contrast, DMD increased markedly in all SMS–OS treatments. This apparent divergence between OMD and DMD likely reflects differences in how these indices capture the fate of lignified residues. SMS consists of partially degraded crop residues enriched with fungal biomass and residual lignocellulolytic enzymes [5],[10],[25]. Fungal pretreatment by *Pleurotus* spp. partially disrupts the lignin–carbohydrate complex, enhancing particle fragility and physical disintegration. Partial delignification and disruption of the lignin–carbohydrate complex by *Pleurotus* spp. can enhance physical disintegration and washout of particles from the bags, increasing DM disappearance even when not all material is fully fermented to VFAs and CO_2_. In addition, the higher EE content of the SMS–OS diets may have contributed to greater DMD through the loss of lipid-rich particles during washing and drying, which are counted as digested DM but do not necessarily increase ruminal OM fermentation.

The modest decline in OMD should therefore be viewed primarily as a consequence of increased lignin content and reduced NFC, rather than as evidence of impaired ruminal fermentation. Importantly, OMD remained above 95% across all diets, values comparable to or exceeding those reported in other studies using fungal-treated residues [9],[26]. Unsaturated lipids from oilseeds are known to partially suppress amylolytic and cellulolytic bacteria and redirect metabolism toward biohydrogenation, thereby reducing acidogenesis without necessarily compromising overall energy extraction [27]–[29]. This mechanism is consistent with the small but significant reduction in OMD at the highest SMS–OS inclusion level alongside stable total VFA concentrations.

The improvement in DMD observed here contrasts with *in vivo* findings by Lu et al. [30], who reported reduced digestibility when SMS exceeded 10% of dietary DM. This discrepancy likely reflects the controlled conditions of the RUSITEC, which eliminate the effects of chewing behavior, selective intake, passage rate, and intake regulation present *in vivo*. Under semi-continuous flow and stable pH, the pretreated nature of SMS appears to facilitate DM disappearance without the physical or kinetic constraints encountered in the live animal.

### 4.3. Volatile fatty acid profiles

Despite substantial changes in diet composition and digestibility, total VFA concentration was affected by SMS–OS inclusion, remaining within physiologically relevant ranges across all treatments. This indicates that overall ruminal fermentation intensity was largely preserved. Because VFAs supply a major proportion of metabolizable energy to ruminants [31],[32], the maintenance of total VFA production suggests that SMS–OS inclusion does not compromise fermentative efficiency under RUSITEC conditions.

The moderate nature of the VFA response aligns well with the observed compositional changes, namely the decline in NFC alongside increases in NDF and EE. This combination of reduced readily fermentable carbohydrate and increased lipid supply is known to temper acid production and stabilize ruminal fermentation patterns rather than markedly decrease VFA output [33]–[35].

Acetate proportion declined with increasing SMS–OS inclusion, with CS60 exhibiting a value approximately 10% lower than that of the control. This tendency is consistent with the higher lignin content, lower NFC availability, and potential inhibitory effects of unsaturated lipids on cellulolytic bacteria such as *Ruminococcus flavefaciens* and *Fibrobacter succinogenes* [36],[37]. The relatively high acetate proportions observed in CS90 and CS80 may reflect improved fiber accessibility resulting from fungal pretreatment [26],[38], whereas the substantially higher ADL content in CS60 may have partially offset these benefits.

The CS60 showed a higher propionate proportion. Propionate serves as an important hydrogen sink and glucogenic precursor [39], and diets enriched with unsaturated fatty acids from oilseeds often promote propionogenic pathways and hydrogen utilization via biohydrogenation [28],[40]. The increase in propionate, together with the lower acetate and the reduced acetate:propionate, is consistent with the marked reductions in CH_4_ production observed at higher SMS–OS inclusion levels.

Butyrate proportion was unaffected by treatment, indicating relative stability of butyrogenic pathways. In contrast, minor VFAs (valerate and isovalerate) responded to SMS–OS inclusion. Valerate concentration increased in CS80 and CS60, and isovalerate proportion in CS60. These branched-chain VFAs, products of amino acid deamination, [41],[42], serve as growth factors for fibrolytic bacteria particularly *Fibrobacter succinogenes*, *Ruminococcus albus*, and *R. flavefaciens* [43] and likely contribute to maintaining NDF digestibility despite elevated lignin content. Their increase at higher SMS–OS inclusion levels likely reflects enhanced silage fermentation of fungal-derived protein from SMS and contributions from oilseed protein. The maintenance of high NDF digestibility despite elevated lignin suggests that these VFAs support fibrolytic activity under more recalcitrant substrate conditions.

### 4.4. Greenhouse gas and ammonia emissions

A major outcome of this study was that some SMS–OS treatments reduced CH_4_ production, although the reductions were not uniform across treatments. Relative to the control, CH_4_ output declined in SMS–OS diets; however, differences were less pronounced when CH_4_ was expressed per gram of degraded DM or total gas production, indicating that mitigation was moderate and treatment-dependent rather than absolute.

These reductions occurred in diets with similar CP content and modestly higher NDF but markedly increased EE and ADL. Lipid supplementation, specifically unsaturated fatty acids from OS, likely contribute substantially to CH_4_ mitigation by acting as alternative hydrogen sinks through biohydrogenation, inhibiting methanogenic archaea and protozoa, and competing with CO_2_ for reducing equivalents [33],[34]. The EE in CS60 fell within the range reported to reduce CH_4_ without strongly depressing fiber digestion [44],[45].

In addition to lipid effects, changes in carbohydrate composition and fiber quality due to SMS inclusion also influenced ruminal fermentation. Although EE increased progressively across treatments, the increase in ADL in CS60 likely limits substrate availability for methanogens, partially offsetting the lipid-mediated reduction in CH_4_ [46]. Higher lignin and lower NFC may have shifted ruminal fermentation toward pathways generating less hydrogen per unit of DM degraded, redistributing hydrogen toward propionate synthesis and microbial protein rather than CH_4_ formation [47],[48]. Differences in hydrogen recovery efficiency among treatments likely contributed to the observed pattern, with CS90 supporting the most favorable partitioning of hydrogen toward propionate and microbial protein synthesis, reducing the substrate available for CH_4_ formation. The CS90 diet may represent an optimal lipid threshold for antimethanogenic action, whereas higher inclusion in CS60, in combination with elevated lignin, may exceed inhibitory thresholds for key fibrolytic bacteria, thereby limiting further reductions in CH_4_. Taken together, the non-linear CH_4_ reduction pattern is attributable to the interaction of lignin content, lipid supplementation, and hydrogen partitioning efficiency, with CS90 achieving the strongest mitigation by combining moderate lignin, sufficient lipid-mediated suppression of methanogens, and optimal hydrogen utilization for propionate and microbial protein synthesis.

The lower acetate:propionate and increased valerate and isovalerate at higher SMS–OS inclusion support a redistribution of reducing equivalents away from methanogenesis. SMS may also contribute directly to CH_4_ mitigation.

*Pleurotus*-based SMS may also contribute directly to CH_4_ modulation. SMS contains phenolic compounds and other secondary metabolites with antimicrobial activity against methanogens [49],[50]. It is important to note that, while reductions in CH_4_ were observed, the magnitude and consistency across metrics were moderate, and total gas production and fiber digestibility were largely maintained. This underscores that SMS-OS inclusion may influence CH_4_ emissions via multiple, interacting mechanisms rather than producing a uniform, large-scale mitigation effect. *In vivo*, the altered physical structure of SMS may also accelerate particle passage and reduce methanogenesis time, although this mechanism cannot be directly assessed in RUSITEC. Studies replacing conventional forages with mushroom-cultivated residues have similarly reported reductions in CH_4_ and shifts in methanogen communities [49],[50], consistent with our findings.

Moreover, CO_2_ productions followed a similar pattern, decreasing in diets containing SMS-OS. Reductions in CO_2_ likely reflect decreased decarboxylation associated with ruminal carbohydrate fermentation and altered fermentation stoichiometry as hydrogen is redirected from CH_4_- and CO_2_-generating pathways. Similar reductions in CO_2_ with SMS inclusion have been reported *in vitro* [7],[11],[51], supporting the notion that co-ensiling with SMS can influence gaseous fermentation products through chemical and microbial mechanisms.

NH_3_ concentration and H_2_S production were not significantly affected by treatment, but both showed consistent numerical declines with SMS–OS inclusion. NH_3_ concentrations were 14–36% lower in CS90–CS60 than in the control, suggesting improved capture of ruminal NH_3_ into microbial protein and reduced deamination. Fungal biomass in SMS provides slowly degradable, cell wall–associated nitrogen that may be better synchronized with carbohydrate fermentation [10],[52], and a fraction of oilseed protein is rumen-undegradable, reducing the pool of rapidly deaminated nitrogen. Similar reductions in ruminal NH_3_ have been observed when *Pleurotus* SMS was included in sheep diets [52]. H_2_S emissions declined numerically by 66–80% with SMS–OS inclusion, although variability prevented statistical significance. Given the complexity of ruminal sulfur metabolism [53], this trend may reflect altered sulfur availability or microbial competition following fungal pretreatment, but the underlying mechanisms require further investigation.

### 4.5. Fiber digestibility

Fiber digestibility data reinforce the CH_4_ mitigation results by demonstrating that NDF digestibility remained high and unaffected by SMS–OS inclusion, despite substantial increases in ADL. Because NDF concentrations differed only slightly among diets, the amount of cell wall substrate available to microbes was broadly comparable. The maintenance of NDF digestibility suggests that the beneficial effects of fungal pretreatment on fiber accessibility offset the inhibitory effects of added lignin and unsaturated lipids. Moreover, *Pleurotus* spp. produce a suite of lignocellulolytic enzymes that disrupt the lignin–carbohydrate complex and increase tissue porosity [10],[25],[26], likely preserving NDF utilization.

ADLD was significantly affected, although slightly higher values in CS90 and CS60 suggest that a portion of SMS lignin is more susceptible to microbial attack than native corn lignin. Selective lignin modification by *Pleurotus* spp. produces a less condensed structure that may be partially degradable in the rumen [26],[38].

## 5. Conclusions

Partial replacement of corn silage with spent mushroom substrate and oilseeds substantially modified silage chemical composition. Under RUSITEC conditions, the compositional changes did not compromise ruminal fermentation stability, as ruminal pH remained within a narrow physiological range, and total gas productions were maintained. Fiber utilization was largely preserved, with neutral detergent fiber digestibility unaffected and only a minor tendency toward reduced acid detergent fiber digestibility at the highest SMS–oilseed inclusion level, despite a marked increase in lignin content. Co-ensiling corn silage with SMS and oilseeds consistently enhanced dry matter digestibility, which increased by approximately 29–37% relative to the control, while organic matter digestibility declined only modestly at the highest replacement level. VFA profiles indicated enhanced overall VFA supply, accompanied by subtle shifts toward lower acetate:propionate at higher SMS–oilseed inclusion levels. Most importantly, all SMS–oilseed treatments substantially reduced greenhouse gas emissions, decreasing CH_4_ production by approximately 35–60% and CO_2_ emissions by 17–49% without impairing fiber digestion or overall fermentative activity. Among the evaluated treatments, CS90 (90% corn silage with 5% SMS and 5% oilseeds) emerged as the most suitable option for practical application. These findings highlight the potential of SMS–oilseed co-ensiling as a climate-smart feeding strategy, warranting further validation under *in vivo* conditions.

## Use of AI tools declaration

The authors declare they have not used Artificial Intelligence (AI) tools in the creation of this article.
